# Hepatoprotective Effect of *Allium ochotense* Extracts on Chronic Alcohol-Induced Fatty Liver and Hepatic Inflammation in C57BL/6 Mice

**DOI:** 10.3390/ijms25063496

**Published:** 2024-03-20

**Authors:** Min Ji Go, Jong Min Kim, Hyo Lim Lee, Tae Yoon Kim, Ju Hui Kim, Han Su Lee, In Young Kim, Seon Jeong Sim, Ho Jin Heo

**Affiliations:** 1 Division of Applied Life Science (BK21), Institute of Agriculture and Life Science, Gyeongsang National University, Jinju 52828, Republic of Korea; alswl9245@gnu.ac.kr (M.J.G.); myrock201@gnu.ac.kr (J.M.K.); gyfla059@gnu.ac.kr (H.L.L.); kty8747@gnu.ac.kr (T.Y.K.); zkfkapflove@nate.com (J.H.K.); ns3005@naver.com (H.S.L.); inzero331@naver.com (I.Y.K.); 2 Forest Research Department, Gyeongsangnam-do Forest Environment Research Institute, Jinju 52615, Republic of Korea; sjsim23@korea.kr

**Keywords:** *Allium ochotense*, alcoholic liver disease, lipid metabolism, alcoholic hepatitis, apoptosis, liver fibrosis

## Abstract

This study was performed to investigate the protective effects of *Allium ochotense* on fatty liver and hepatitis in chronic alcohol-induced hepatotoxicity. The physiological compounds of a mixture of aqueous and 60% ethanol (2:8, *w*/*w*) extracts of *A. ochotense* (EA) were identified as kestose, raffinose, kaempferol and quercetin glucoside, and kaempferol di-glucoside by UPLC Q-TOF MS^E^. The EA regulated the levels of lipid metabolism-related biomarkers such as total cholesterol, triglyceride, low-density lipoprotein (LDL), and high-density lipoprotein (HDL)-cholesterol in serum. Also, EA ameliorated the levels of liver toxicity-related biomarkers such as glutamic oxaloacetic transaminase (GOT), glutamic pyruvic transaminase (GPT), and total bilirubin in serum. EA improved the antioxidant system by reducing malondialdehyde contents and increasing superoxide dismutase (SOD) levels and reduced glutathione content. EA improved the alcohol metabolizing enzymes such as alcohol dehydrogenase, acetaldehyde dehydrogenase, and cytochrome P450 2E1 (CYP2E1). Treatment with EA alleviated lipid accumulation-related protein expression by improving phosphorylation of AMP-activated protein kinase (p-AMPK) expression levels. Especially, EA reduced inflammatory response by regulating the toll-like receptor–4/nuclear factor kappa-light-chain-enhancer of activated B cells (TLR-4/NF-κB) signaling pathway. EA showed an anti-apoptotic effect by regulating the expression levels of B-cell lymphoma 2 (BCl-2), BCl-2-associated X protein (BAX), and caspase 3. Treatment with EA also ameliorated liver fibrosis via inhibition of transforming growth factor-beta 1/suppressor of mothers against decapentaplegic (TGF-β1/Smad) pathway and alpha-smooth muscle actin (α-SMA). Therefore, these results suggest that EA might be a potential prophylactic agent for the treatment of alcoholic liver disease.

## 1. Introduction

Excessive consumption of alcohol can be a cause of social, economic, and clinical complications worldwide, and alcoholic liver disease (ALD) is regarded as a leading cause of alcohol-related death [1,2]. Excessive drinking over a long period of time can cause damage to most organs of the body, and rapid deterioration of the liver, an essential part of ethanol metabolism, can be fatal [1]. Alcohol, which is rapidly absorbed from the gastrointestinal tract and metabolized by hepatocytes by alcohol dehydrogenase (ADH) into acetaldehyde, is a highly toxic compound [2]. It is then further metabolized into acetate by acetaldehyde dehydrogenase (ALDH), and with chronic consumption of alcohol, expression of cytochrome P4502E1 (CYP2E1) enzyme can show a notable increase, leading to the production of reactive oxygen species (ROS), oxidative stress, and inflammation [3]. Although the pathogenesis of ALD has not yet been clearly determined, it is believed to include lipid peroxidation, oxidative stress, production of ROS, and metabolic disorders of the liver in addition to the direct hepatotoxicity of ethanol [3]. In addition, acetaldehyde produced during the metabolism of ethanol can increase the ratio of reduced nicotinamide adenine dinucleotide (NADH) and oxidized nicotinamide adenine dinucleotide (NAD^+^), thereby reducing β-oxidation of fatty acids, thereby inducing fatty liver [4]. In particular, alcohol and/or acetaldehyde can inhibit the phosphorylation of AMP-activated protein kinase (AMPK) and increase the expression of sterol regulatory element-binding proteins-1 (SREBP-1), resulting in an imbalance in lipid metabolism, ultimately contributing to the development of hepatic steatosis [4,5]. Accumulation of lipids in hepatocytes can be classified as a characteristic of early ALD and may progress to alcoholic steatohepatitis characterized by liver cell damage and inflammation of liver cells or acute alcoholic hepatitis [1,2,6]. Also, increased intestinal permeability due to excessive consumption of ethanol can increase the transport of Gram-negative bacterial-derived lipopolysaccharide (LPS) from the intestine to the liver [1]. It can induce activation of Kupffer cells through the LPS/toll-like receptor–4 (TLR-4) pathway, resulting in activation of nuclear factor kappa-light-chain-enhancer of activated B cells (NF-κB), with the production of pro-inflammatory cytokines including tumor necrosis factor-alpha (TNF-α) [1,4]. In addition, increased levels of pro-inflammatory cytokines can accelerate the formation of free radicals through a reduction of nicotinamide adenine dinucleotide phosphate (NADPH) oxidase and induction of CYP2E1 [1]. Production of ROS and oxidative stress caused by ethanol or acetaldehyde can increase the translocation of B-cell lymphoma 2 (BCl-2)-associated X protein (BAX) to the outer mitochondrial membrane [7]. Thus, mitochondrial membrane permeability and transition potential are reduced, and the release of cytochrome C can lead to the activation of factors associated with the endogenous apoptotic pathway such as caspase 9 and caspase 3 [6]. Liver damage can occur in ALD patients; acetaldehyde-protein adducts can cause damage to hepatocyte mitochondria but can also activate stellate cells along with inflammatory cells in liver sinusoids [2,3]. Activated stellate cells can induce fibrotic genes such as alpha-1 smooth muscle actin and collagen-1 and produce collagen. Sustained and long-term activation of hepatic stellate cells and myofibroblasts can ultimately lead to the development of liver fibrosis [2]. Cirrhosis, characterized by liver fibrosis, is irreversible. Therefore, early prevention of ALD when no irreversible injury has occurred is important [8].

*Allium ochotense* (*A. ochotense*) is a Korean endemic species with distribution on Jiri Mountain and Seorak Mountain [9]. The genus *Allium* can be classified according to approximately 20 species. *Allium victorialis* (*A. victorialis*), which shares similar biological characteristics with *A. ochotense*, belongs to the Liliaceae family and has a wide distribution in northern Korea [10]. Previous studies have reported that the extract from this plant can exhibit anti-hyperlipidemic, anti-obesity, and anti-hepatotoxic activity, and contains flavonoids, steroid saponins, and sulfur compounds [9,10]. In particular, one study reported that *A. ochotense* leaves are rich in flavonoids including kaempferol, quercetin, and astragalin, as well as phenolic compounds such as ferulic acid [11]. Contrary to such reports, knowledge regarding the mechanism of the protective effect of *A. ochotense* extract against alcohol-induced liver toxicity is limited. Findings from our previous study confirmed the in vitro antioxidant and cytoprotective effects of *A. ochotense* extract against ethanol-induced hepatocyte toxicity using *A. ochotense* extract [11]. Based on these preliminary findings, *A. ochotense* extract was used in this study to examine the mechanism of the effect of improvement of lipid metabolism disorders and alcoholic hepatitis in the liver of alcohol-induced experimental animals.

## 2. Results

### 2.1. The Activity of Alcohol Degradation in Serum

**Figure 1:**
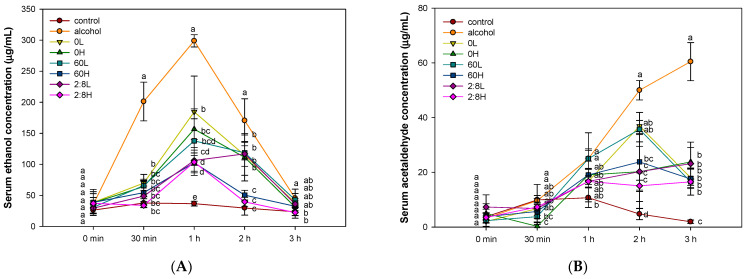

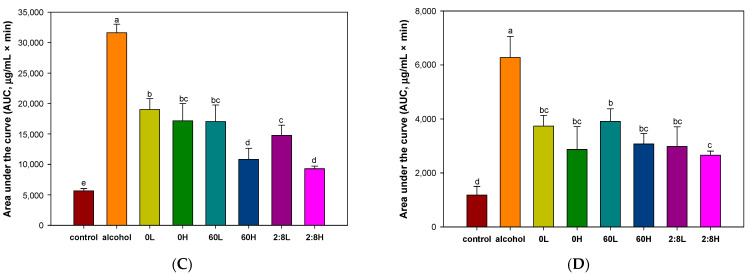
Effect of the aqueous extract, 60% ethanolic extract of *Allium ochotense*, and the mixture of aqueous and 60% ethanol (2:8, *w*/*w*) extracts of *Allium ochotense* (EA) on ethanol and acetaldehyde concentration in serum. (**A**) Serum ethanol concentration, (**B**) acetaldehyde concentration, (**C**) area under the curve (AUC) of ethanol concentration, and (**D**) AUC of acetaldehyde concentration. 0 L, the aqueous extract of *Allium ochotense* (50 mg/kg of B.W.) + alcohol; 0 H, the aqueous extract of *Allium ochotense* (100 mg/kg of B.W.) + alcohol; 60 L, the 60% ethanol extract of *Allium ochotense* (50 mg/kg of B.W.) + alcohol; 60 H, the 60% ethanol extract of *Allium ochotense* (100 mg/kg of B.W.) + alcohol; 2:8 L, the mixture of aqueous and 60% ethanol extracts of *Allium ochotense* (50 mg/kg of B.W.) + alcohol; 2:8 H, the mixture of aqueous and 60% ethanol extract of *Allium ochotense* (100 mg/kg of B.W.) + alcohol. The results are shown as mean ± SD (*n* = 3). Data were statistically considered at *p* < 0.05, and different lowercase letters indicate statistical differences.

Alcohol and acetaldehyde concentrations and each concentration over time in the serum were detected to evaluate alcohol degradation metabolism. As shown in Figure 1, concerning the alcohol concentration in serum, there was no significant difference between each group at 0 min. At points of all time after 30 min, the ethanol concentrations in the serum of the alcohol group (201.18, 298.92, 170.43, and 45.53 µg/mL, respectively, over time) were significantly increased over that of the control group (37.71, 36.69, 29.70, and 23.67 µg/mL, respectively, over time). On the other hand, the high-concentration (100 mg/kg of body weight (B.W.)) treatment group of a mixture of *A. ochotense* extract (33.84, 103.63, 39.65 and 22.40 µg, respectively, over time) showed a similar level of alcohol concentration as the control group at 2 h and 3 h after alcohol exposure (Figure 1A). In addition, the area under the curve (AUC) of ethanol concentration showed that the alcohol group (31,609.08 μg/mL × min) was significantly increased compared to the control group (5668.72 μg/mL × min) (Figure 1C). The high-concentration treatment group of the mixture of *A. ochotense* extract (9287.57 μg/mL × min) showed the lowest total concentration compared to the other extract treatment groups.

In addition, in the evaluation of acetaldehyde concentration, there was no significant difference between each group at 0 min (Figure 1B). After 1 h of alcohol exposure, the acetaldehyde concentration of the alcohol group (25.00, 50.00, and 60.45 µg/mL, respectively, over time) significantly increased compared to the control group (10.71, 4.76, and 1.93 µg/mL, respectively, over time). On the other hand, at 180 min after alcohol exposure, all the *A. ochotense* extract treatment groups significantly improved the increased blood acetaldehyde concentration in the alcohol group. Additionally, the AUC of acetaldehyde concentration showed that the alcohol group (6275.49 μg/mL × min) was significantly increased compared to the control group (1180.22 μg/mL × min) (Figure 1D). In particular, the high-concentration treatment group of the mixture of *A. ochotense* extract (2659.37 μg/mL × min) showed the lowest blood acetaldehyde concentration compared to other treatment groups.

In summary, the mixture of *Allium ochotense* extract was shown to help improve the alcohol degradation ability more than every single extract. Therefore, the protective effect against alcoholic fatty liver induced by chronic alcohol exposure was evaluated using the mixture of aqueous and 60% ethanolic extracts of *Allium ochotense* (EA).

### 2.2. Biologically Active Substances in the Mixture of Aqueous and 60% Ethanol Extracts of Allium ochotense (EA)

**Figure 2:**
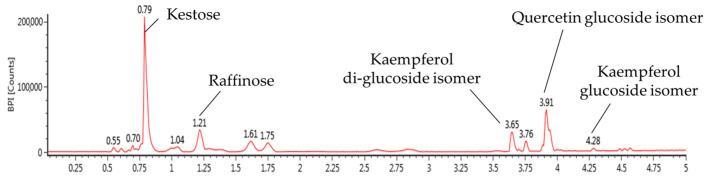
UPLC-Q-TOF/MS^E^ chromatography in negative ion mode in the mixture of aqueous and 60% ethanol (2:8, *w*/*w*) extracts of *Allium ochotense* (EA).

The results of identifying EA using ultra-performance liquid chromatography-quadrupole time of flight mass spectrometry (UPLC-QTOF/MS^E^) analysis are shown in Figure 2 and Table 1. The results of compound estimation and identification compared to MS fragments from previous studies are as follows: kestose (retention time (RT), 0.79 min and *m*/*z*, 503; 179, 341, 101, 323, 221, 113), raffinose (RT, 1.12 min and *m*/*z* 503; 341, 323, 211, 179, 143), kaempferol di-glucoside isomer (RT, 3.65 min and *m*/*z* 609; 447, 285), quercetin glucoside isomer (RT, 3.91 min and *m*/*z* 463; 301, 285, 464), kaempferol glucoside isomer (RT, 4.28 min and *m*/*z* 447; 174, 285, 229).

### 2.3. The Regulation of Fatty Liver and Hepatotoxicity Biomarkers in Serum

Table 2 shows the results of measuring the effect of EA on biomarkers related to alcohol-induced lipid metabolism imbalance. As a result of measuring levels of the total cholesterol (TCHO), triglycerides (TG), and low-density lipoprotein (LDL)-cholesterol, the alcohol group (110.25 mg/dL, 143.50, and 11.40 mg/dL, respectively) was significantly increased compared to the control group (94.50, 115.50, and 3.45 mg/dL, respectively) and the normal sample (NS) group (95.75 mg/dL, 101.75 mg/dL, and 4.00 mg/dL). On the other hand, the high-concentration (100 mg/kg of B.W.) treatment group of the mixture of *A. ochotense* extracts (EA100; 105.00, 113.25, 0.55 mg/dL, respectively) decreased significantly compared to the alcohol group. In contrast to these results, high-density lipoprotein (HDL)-cholesterol levels were significantly reduced in the alcohol group (70.50 mg/dL) compared to the control group (74.00 mg/dL) and NS group (76.25 mg/dL). The EA100 (91.00 mg/dL) significantly increased compared to the alcohol group.

Table 2 shows the results of measuring the effect of the mixture of *A. ochotense* extract on alcohol-induced liver toxicity biomarkers. As a result of measuring levels of GOT and GPT, the alcohol group (54.00 U/L and 35.40 U/L, respectively) was significantly increased compared to the control group (45.40 U/L and 29.40 U/L, respectively) and NS group (44.80 U/L and 29.20 U/L, respectively). On the other hand, EA100 (46.00 U/L and 29.40 U/L, respectively) significantly improved the alcohol-induced increase of the levels of GOT and GPT. Total bilirubin levels were shown to be significantly increased in the alcohol group (0.70 mg/dL) compared to the control group (0.50 mg/dL) and NS group (0.52 mg/dL). Regarding these results, the low-concentration (50 mg/kg of B.W.) treatment group of the mixture of *A. ochotense* extracts (EA50; 0.38 mg/dL) and EA100 (0.42 mg/dL) improved the total bilirubin level to a similar level as the control group.

### 2.4. The Activity of Antioxidant System in Liver Tissue

**Figure 3:**
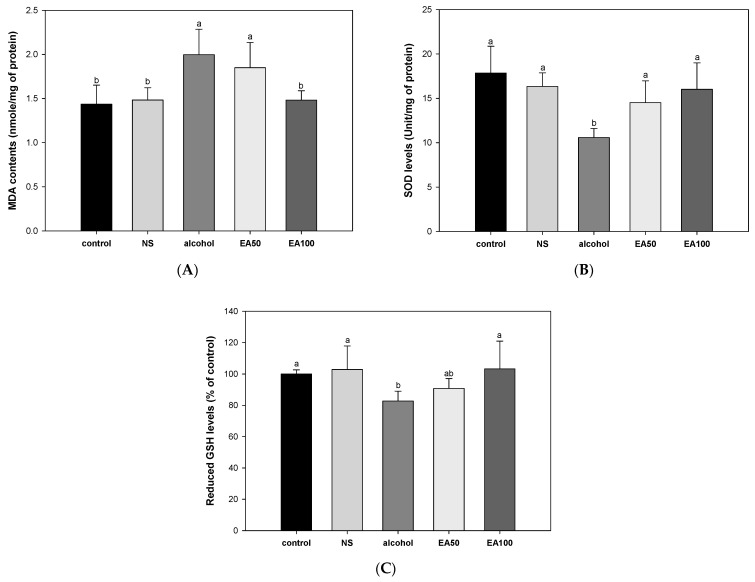
Ameliorating effect of the mixture of aqueous and 60% ethanol (2:8, *w*/*w*) extracts of *Allium ochotense* (EA) on the antioxidant system. (**A**) The malondialdehyde (MDA) contents, (**B**) superoxide dismutase (SOD) levels, and (**C**) reduced glutathione (GSH) levels. NS group, the mixture of aqueous and 60% ethanol extracts of *Allium ochotense* (100 mg/kg of B.W.) + drinking water; EA50 group, the mixture of aqueous and 60% ethanol extracts of *Allium ochotense* (50 mg/kg of B.W.) + alcohol; EA100 group, the mixture of aqueous and 60% ethanol extracts of *Allium ochotense* (100 mg/kg of B.W.) + alcohol. The results are shown as mean ± SD (ex vivo test, *n* = 7). Data were statistically considered at *p* < 0.05, and different lowercase letters indicate statistical differences.

The results of investigating the improvement effect of EA on alcohol-induced liver antioxidant system imbalance are shown in Figure 3. The results of measuring the effect of *Allium ochotense* on malondialdehyde (MDA) content are shown in Figure 3A. The alcohol group (2.00 nmol/mg of protein) had a significantly increased MDA content compared to the control group (1.44 nmol/mg of protein) and the NS group (1.48 nmol/mg of protein). On the other hand, EA100 (1.48 nmol/mg of protein) improved the alcohol-induced increase in levels of MDA similar to that of the control group.

The results of measuring the effect of EA on superoxide dismutase (SOD) levels are shown in Figure 3B. The SOD level of the alcohol group (10.59 Unit/mg of protein) was significantly reduced compared to the control group (17.85 Unit/mg of protein) and the NS group (16.34 Unit/mg of protein). In contrast to these results, EA50 (14.52 Unit/mg of protein) and EA100 (16.02 Unit/mg of protein) groups significantly improved this. In particular, EA100 was confirmed to improve to a similar level as the control group.

The results of measuring the effect of EA on reduced glutathione (GSH) levels are shown in Figure 3C. The level of reduced GSH in the alcohol group (82.78% of control) was significantly decreased compared to the control group (100%) and NS group (102.92% of control). On the other hand, EA100 (103.25% of control) improved to a similar level as the control group.

### 2.5. The Activity of Extracted Mitochondrial Function in Liver Tissue

**Figure 4:**
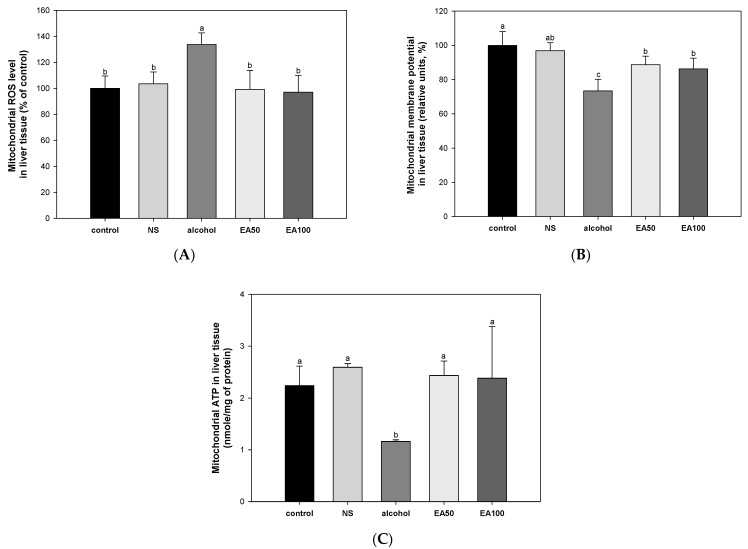
Effect of the mixture of aqueous and 60% ethanol (2:8, *w*/*w*) extracts of *Allium ochotense* (EA) on mitochondrial activity. (**A**) The mitochondrial reactive oxygen species (ROS), (**B**) mitochondrial membrane potential (MMP) levels, and (**C**) mitochondrial ATP levels. NS group, the mixture of aqueous and 60% ethanol extracts of *Allium ochotense* (100 mg/kg of B.W.) + drinking water; EA50 group, the mixture of aqueous and 60% ethanol extracts of *Allium ochotense* (50 mg/kg of B.W.) + alcohol; EA100 group, the mixture of aqueous and 60% ethanol extracts of *Allium ochotense* (100 mg/kg of B.W.) + alcohol. The results are shown as mean ± SD (mitochondrial analysis, *n* = 5). Data were statistically considered at *p* < 0.05, and different lowercase letters indicate statistical differences.

The results of investigating the improvement effect of the mixture of EA on alcohol-induced liver mitochondrial dysfunction are shown in Figure 4. As a result of measuring the ROS content in mitochondria, the alcohol group (133.63% of control) showed a significant increase compared to the control group (100%) and the NS group (103.44% of control) (Figure 4A). On the other hand, EA50 (99.17% of control) and EA100 (96.96% of control) groups improved oxidative stress in mitochondria to a similar level as the control group.

As a result of evaluating the mitochondrial membrane potential (MMP) level, the alcohol group (73.42%) showed a significantly reduced level compared to the control group (100%) and NS (96.93%) (Figure 4B). EA50 (88.69%) and EA100 (86.23%) groups significantly increased the reduced membrane potential level and significantly improved compared to the alcohol group.

The evaluation of mitochondrial ATP levels showed a significant decrease in the alcohol group (1.16 nmole/mg of protein) compared to the control group (2.24 nmole/mg of protein) and NS (2.59 nmole/mg of protein) (Figure 4C). On the other hand, EA50 (2.44 nmole/mg of protein) and EA100 (2.38 nmole/mg of protein) significantly increased compared to the alcohol group and improved to a similar level as the control group.

### 2.6. The Expression Levels of Alcohol Degradation Enzyme Related Biomarkers

**Figure 5:**
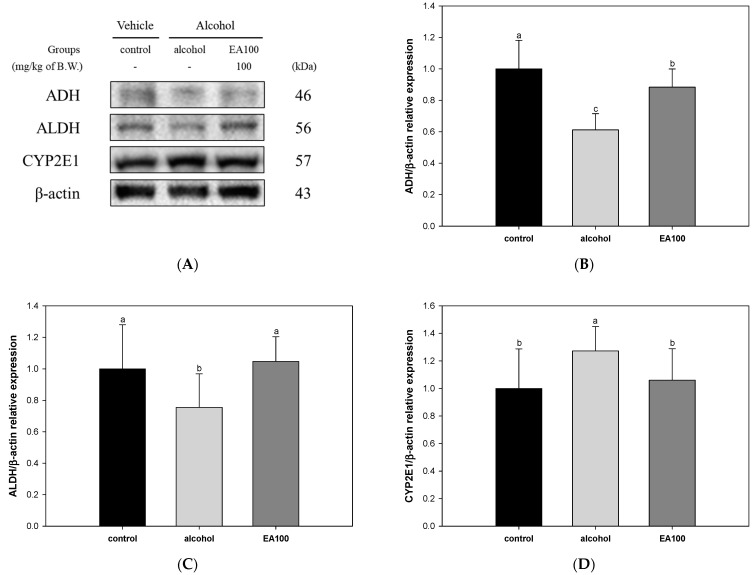
Ameliorating effect of the mixture of aqueous and 60% ethanol (2:8, *w*/*w*) extracts of *Allium ochotense* (EA) on alcohol-metabolizing enzymes in liver tissue. (**A**) The western blot membrane image; the expression levels of (**B**) alcohol dehydrogenase (ADH), (**C**) acetaldehyde dehydrogenase (ALDH), and (**D**) cytochrome P450 2E1 (CYP2E1). EA100 group, the mixture of aqueous and 60% ethanol extracts of *Allium ochotense* (100 mg/kg of B.W.) + alcohol. The results are shown as mean ± SD (western blot analysis, *n* = 3). Data were statistically considered at *p* < 0.05, and different lowercase letters indicate statistical differences.

The results of an experiment to evaluate the alcohol degradation ability mechanism of the EA in the alcohol-induced liver and the results of measuring alcohol decomposition enzymes ADH, ALDH, and CYP2E1 are shown in Figure 5. The expression levels of ADH and ALDH, which are alcohol-degrading enzymes, were shown to be significantly reduced in the alcohol group (0.61 and 0.76, respectively) compared to the control group, and EA100 (0.88 and 1.05, respectively) significantly improved the expression levels (Figure 5B,C). On the other hand, the expression level of CYP2E1 in the alcohol group (1.27) was significantly increased compared to the control group, and EA100 (1.06) significantly decreased compared to the alcohol group (Figure 5D).

### 2.7. The Expression Levels of Lipid Metabolism Related Biomarkers

**Figure 6:**
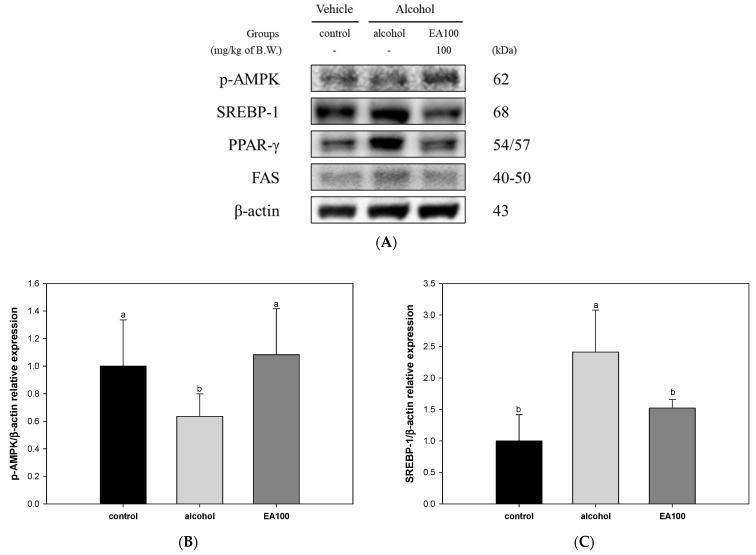

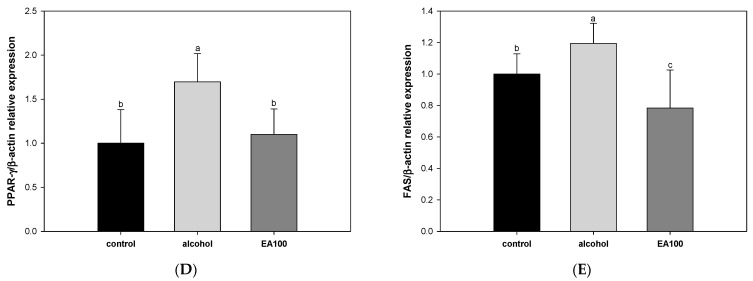
Regulating effect of the mixture of aqueous and 60% ethanol (2:8, *w*/*w*) extracts of *Allium ochotense* (EA) on lipid metabolism in liver tissue. (**A**) The western blot membrane image, and the expression levels of (**B**) phosphorylation of AMP-activated protein kinase (p-AMPK), (**C**) sterol regulatory element-binding proteins-1 (SREBP-1), (**D**) peroxisome proliferator-activated receptor-gamma (PPAR-γ), and (**E**) fatty acid synthase (FAS). EA100 group, the mixture of aqueous and 60% ethanol extracts of *Allium ochotense* (100 mg/kg of B.W.) + alcohol. The results are shown as mean ± SD (western blot analysis, *n* = 3). Data were statistically considered at *p* < 0.05, and different lowercase letters indicate statistical differences.

The results of an experiment to investigate the improvement mechanism against lipid metabolism imbalance of EA on alcoholic fatty liver disease (AFLD) are shown in Figure 6. The expression level of p-AMPK was shown to be significantly decreased in the alcohol group (0.64) compared to the control group. On the other hand, EA100 (1.08) significantly increased compared to the alcohol group (Figure 6B). In addition, the expression levels of SREBP-1, peroxisome proliferator-activated receptor-gamma (PPAR-γ), and fatty acid synthase (FAS) in the alcohol group (2.41, 1.70, and 1.19, respectively) were significantly increased compared to the control group. However, EA100 (1.52, 1.10, and 0.78, respectively) significantly improved alcohol-induced lipid metabolism imbalance compared to the alcohol group (Figure 6C–E).

### 2.8. The Expression Levels of Alcoholic Hepatitis Related Biomarkers

**Figure 7:**
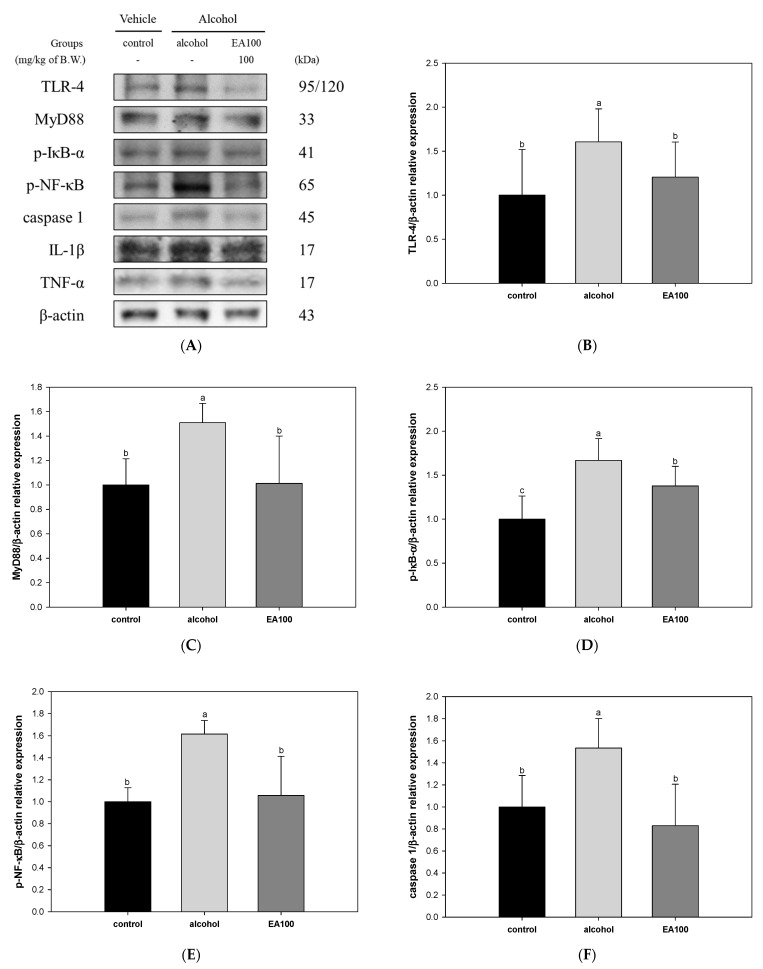

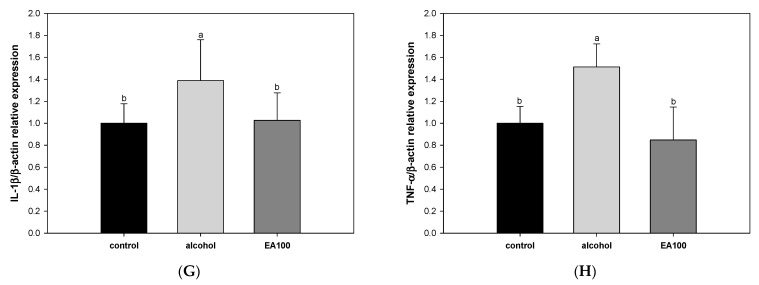
Regulating effect of the mixture of aqueous and 60% ethanol (2:8, *w*/*w*) extracts of *Allium ochotense* (EA) on alcoholic hepatitis in liver tissue. (**A**) The western blot membrane image; the expression levels of (**B**) toll-like receptor–4 (TLR-4), (**C**) myeloid differentiation primary response 88 (MyD88), (**D**) phospho-nuclear factor of kappa light polypeptide gene enhancer in B-cells inhibitor-alpha (p-IκB-α), (**E**) phospho-nuclear factor kappa-light-chain-enhancer of activated B cells (p-NF-κB), (**F**) caspase 1, (**G**) interleukin-1 beta (IL-1β), and (**H**) tumor necrosis factor-alpha (TNF-α). EA100 group, the mixture of aqueous and 60% ethanol extracts of *Allium ochotense* (100 mg/kg of B.W.) + alcohol. The results are shown as mean ± SD (western blot analysis, *n* = 3). Data were statistically considered at *p* < 0.05, and different lowercase letters indicate statistical differences.

The results of investigating the improvement mechanism of alcoholic hepatitis of EA on AFLD are shown in Figure 7. The expression levels of TLR-4 and myeloid differentiation primary response 88 (MyD88) were significantly increased in the alcohol group (1.61 and 1.51, respectively) compared to the control group. On the other hand, EA100 (1.21 and 1.01, respectively) was significantly decreased compared to the alcohol group (Figure 7B,C). In addition, expression levels of a phospho-nuclear factor of kappa light polypeptide gene enhancer in B-cells inhibitor-alpha (p-IκB-α) and p-NF-κB, were significantly increased in the alcohol group (1.62 and 1.67, respectively) compared to the control group and significantly decreased in EA100 (1.06 and 1.38, respectively) (Figure 7D,E). Similarly, the expression levels of caspase 1, interleukin-1 beta (IL-1β), and TNF-α were also significantly increased in the alcohol group (1.54, 1.39, and 1.51, respectively) compared to the control group. In contrast to these results, EA100 (respectively 0.83, 1.03, and 0.85) was significantly improved compared to the alcohol group (Figure 7F–H).

### 2.9. The Expression Levels of Alcohol-Induced Hepatocytes Apoptosis-Related Biomarker

**Figure 8:**
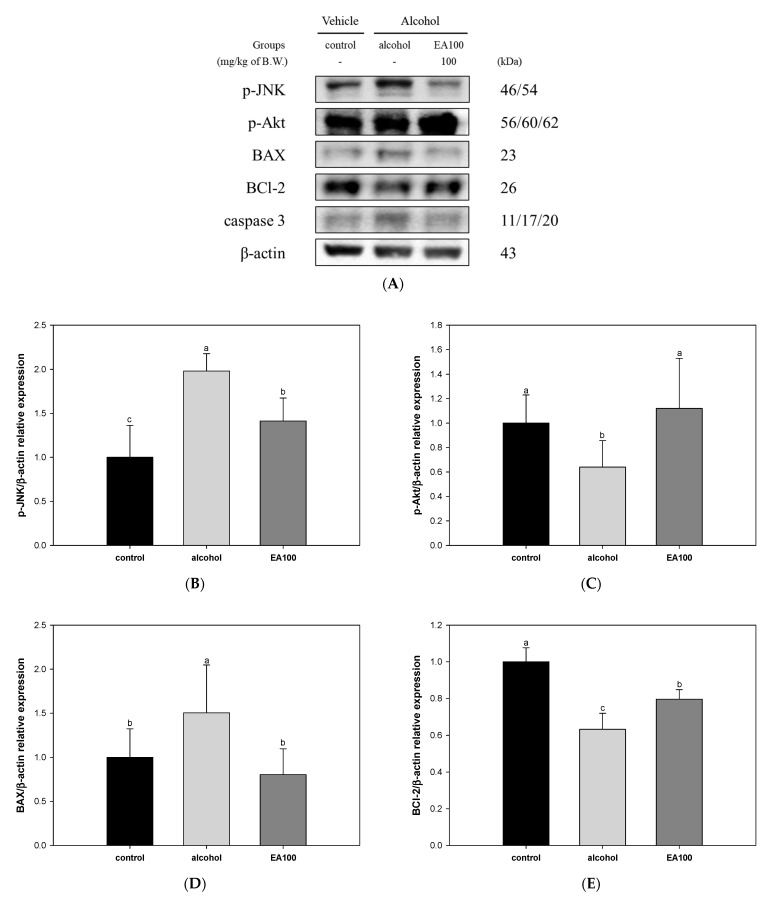

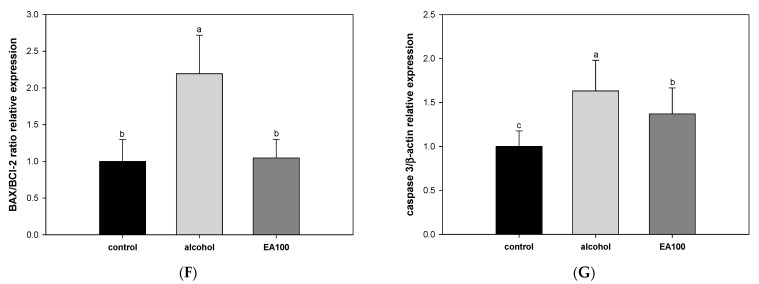
Regulating effect of the mixture of aqueous and 60% ethanol (2:8, *w*/*w*) extracts of *Allium ochotense* (EA) on alcohol-induced hepatocyte apoptosis in liver tissue. (**A**) The western blot membrane image; the expression levels of (**B**) phospho-c-Jun N-terminal kinases (p-JNK), (**C**) phospho-protein kinase B (p-Akt), (**D**) B-cell lymphoma 2 (BCl-2)-associated X protein (BAX), (**E**) BCl-2, (**F**) BAX/BCl-2 ratio, and (**G**) caspase 3. EA100 group, the mixture of aqueous and 60% ethanol extracts of *Allium ochotense* (100 mg/kg of B.W.) + alcohol. The results are shown as mean ± SD (western blot analysis, *n* = 3). Data were statistically considered at *p* < 0.05, and different lowercase letters indicate statistical differences.

The results of investigating the ameliorating mechanism of EA against apoptosis in AFLD are shown in Figure 8. The expression levels of phospho-c-Jun N-terminal kinases (p-JNK) and BAX related to apoptosis, were significantly increased in the alcohol group (1.98 and 1.50, respectively) compared to the control group (Figure 8B,D). EA100 (1.41 and 0.80, respectively) significantly improved compared to the alcohol group. On the other hand, the expression levels of phospho-protein kinase B (p-Akt) and B-cell lymphoma 2 (BCl-2), factors related to anti-apoptosis, were significantly decreased in the alcohol group (0.64 and 0.63, respectively) compared to the control group, and EA100 (1.12 and 0.80, respectively) was significantly improved compared to the alcohol group (Figure 8C,E). As a result of measuring the BAX/BCl-2 ratio relative expression, the BAX/BCl-2 level in the alcohol group (2.19) was shown to be significantly increased compared to the control group. EA100 (1.05) was improved to the expression level similar to the control group (Figure 8F). In addition, the expression level of caspase 3 in the alcohol group (1.16) was significantly increased compared to the control group (Figure 8G). On the other hand, EA100 (1.37) significantly improved compared to the alcohol group.

### 2.10. The Expression Levels of Liver Fibrosis Related Biomarkers

**Figure 9:**
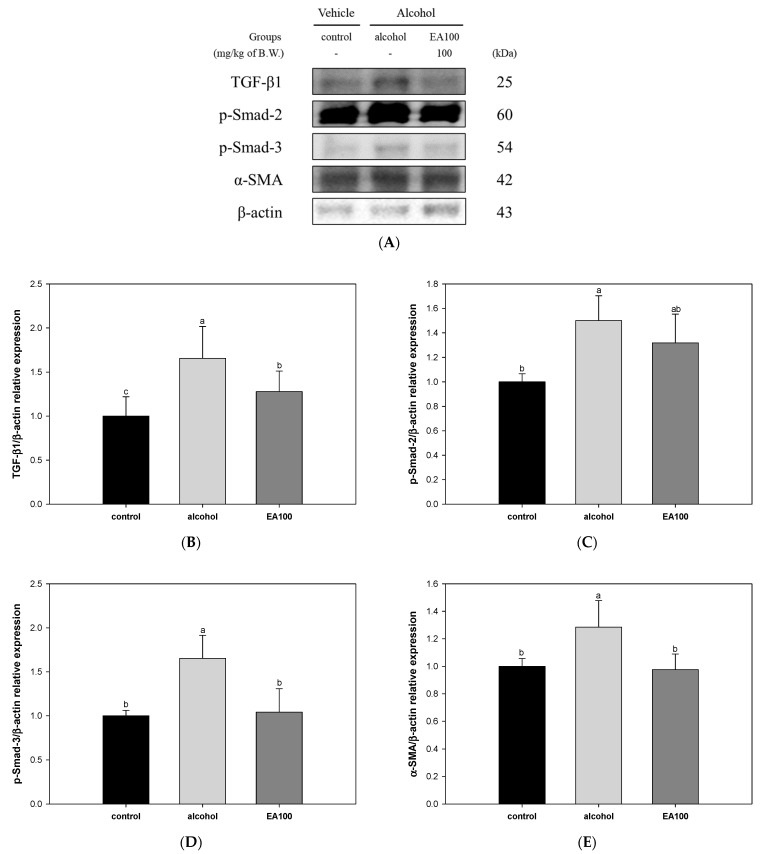
Ameliorating effect of the mixture of aqueous and 60% ethanol (2:8, *w*/*w*) extracts of *Allium ochotense* (EA) on liver fibrosis in fatty liver tissue. (**A**) The western blot membrane image; the expression levels of (**B**) transforming growth factor-beta 1 (TGF-β1), (**C**) phospho-suppressor of mothers against decapentaplegic (p-Smad)-2), (**D**) p-Smad-3, and (**E**) alpha-smooth muscle actin (α-SMA). EA100 group, the mixture of aqueous and 60% ethanol extracts of *Allium ochotense* (100 mg/kg of B.W.) + alcohol. The results are shown as mean ± SD (western blot analysis, *n* = 3). Data were statistically considered at *p* < 0.05, and different lowercase letters indicate statistical differences.

The results of investigating the improvement mechanism of EA on liver fibrosis induced by chronic alcohol are shown in Figure 9. The expression level of transforming growth factor-beta 1 (TGF-β1) was significantly increased in the alcohol group (1.66) compared to the control group. In contrast to these results, EA100 (1.28) significantly improved compared to the alcohol group (Figure 9B). Also, as a result of measuring the expression level of phospho-suppressor of mothers against decapentaplegic-2, 3 (p-Smad-2, 3), they were significantly increased in the alcohol group (1.50 and 1.65, respectively) compared to the control group (Figure 9C,D). On the other hand, EA100 (1.32 and 1.04, respectively) showed improvement compared to the alcohol group. The expression level of alpha-smooth muscle actin (α-SMA) was confirmed to be significantly increased in the alcohol group (1.29) compared to the control group, and EA100 (0.98) was significantly improved compared to the alcohol group (Figure 9E).

## 3. Discussion

Excessive and chronic consumption of alcohol can affect a variety of physiological systems, particularly the liver, the organ responsible for the metabolism of alcohol, along with ALD, including fatty liver, alcoholic steatohepatitis, alcoholic hepatitis, and cirrhosis [6]. However, to date, knowledge regarding the pathogenesis of ALD has been limited, and the hepatotoxicity of ethanol itself, as well as lipid peroxidation, oxidative stress, liver metabolic disorders, and inflammatory response, have been reported as the main pathogenesis [3]. In particular, acetaldehyde, a toxic compound produced during alcohol metabolism, and oxidative stress can be a cause of lipid metabolism disorders [2,4]. Lipid accumulation in liver cells can be classified as early ALD, with development into alcoholic steatohepatitis or alcoholic hepatitis [2,6]. In addition, inflammatory cells can induce activation of hepatic stellate cells and myofibroblasts, leading to cirrhosis characterized by liver fibrosis, which is irreversible, thus recognizing and preventing the disease in early ALD is important [8].

*A. ochotense* leaves have been reported to be rich in flavonoids including kaempferol, quercetin, and astragalin, as well as phenolic compounds such as ferulic acid [11]. Kestose and raffinose, kaempferol di-glucoside isomer, quercetin glucoside isomer, and kaempferol glucoside isomer have also been identified in EA (Figure 2 and Table 1). However, knowledge regarding the mechanism of the protective effect of *A. ochotense* against alcohol-induced liver toxicity is limited. Therefore, in this study, we examined the mechanism of the protective effect against chronic alcohol-induced lipid metabolism disorders and alcoholic hepatitis using *A. ochotense*.

Alcohol, which is absorbed into the blood circulation through the gastrointestinal tract, is then metabolized primarily by hepatocytes. In brief, alcohol is metabolized within hepatocytes by three main enzymes [3]. First, ethanol is mainly metabolized by the ADH enzyme into acetaldehyde in hepatocytes. Acetaldehyde is then converted to acetate using NAD^+^ as a cofactor [6]. The second is the microsomal ethanol-oxidizing system (MEOS) of the smooth endoplasmic reticulum, which becomes more prominent in cases of chronic alcohol consumption. In this case, the CYP2E1 enzyme was required for oxidizing ethanol to acetaldehyde, leading to increased levels of ROS, oxidative stress, and inflammation [3]. The third one is metabolized by catalase (CAT) from peroxisome, which is also an enzyme capable of oxidizing ethanol to acetaldehyde [3]. However, chronic consumption of alcohol can reduce the oxidation of acetaldehyde in healthy mitochondria and reduce the activity of ALDH in liver mitochondria destroyed by deoxycholate, leading to the accumulation of toxic acetaldehyde in the liver [12]. In particular, acetaldehyde produced by alcohol degradation enzymes, which can be highly toxic and reactive, can form covalent bonds with proteins, lipids, and nucleic acids to form acetaldehyde adducts, which can affect the structure and function of macromolecules [1]. In addition, because the induction of hepatic activity of ADH is also inhibited after chronic consumption of alcohol, the generation of ROS through the metabolism of CYP2E1 can mediate liver damage, including fibrosis and cirrhosis, in ALD [12]. Therefore, we attempted to determine whether EA could improve the alcohol degradation ability for acute and chronic alcohol (Figure 1 and Figure 5). According to the results, the blood ethanol and acetaldehyde concentrations of the EA group against acute alcohol decreased over time compared to the alcohol group (Figure 1). In addition, treatment with EA improved the alcohol-induced decrease in levels of ADH and ALDH expression, thereby regulating the expression level of CYP2E1, which is activated chronically and at high ethanol concentrations (Figure 5). According to a previous in vitro study, treatment with *A. ochotense* extract appeared to influence the increased ADH activity rather than ALDH. In particular, arginine detected in *A. ochotense* appeared to influence the activity of enzymes related to alcohol metabolism [11]. In addition, the leaves of *Hovenia Dulcis* contain flavonol tri-glycosides such as kaempferol and quercetin, and the plant extract has been reported to reduce the levels of blood alcohol, promote elimination of alcohol, and prevent dysfunction associated with alcohol abuse [13]. In addition, methyl ferulic acid showed increased activity of hepatic ADH and ALDH in alcohol-induced mice and its hepatoprotective effect from the promotion of alcohol metabolism by reducing the expression of CYP2E1 [14]. Based on these findings, treatment with *A. ochotense* enhanced the activities of ADH and ALDH enzymes and reduced the accumulation of acetaldehyde by promoting the degradation of alcohol. Our findings also suggested that regulating the expression of CYP2E1 can be helpful in improving the decline of alcohol-induced liver function by reducing the production of ROS.

Oxidative stress caused by the production of highly reactive ROS in liver cells can play an important role in the progression of ALD [15]. In particular, upregulation of CYP2E1 due to chronic consumption of alcohol can reduce the levels of antioxidant systems in the body such as SOD and GSH due to the production of high levels of ROS [2,6]. The reaction between ROS and lipid species can be a cause of lipid peroxidation, with the production of lipid peroxides such as 4-hydroxynonenal and MDA. These compounds can cause ferroptosis, a type of iron-dependent programmed cell death, and cell damage and various types of cell death have been reported [6]. In particular, the deposition of MDA can contribute to the accelerated deterioration of damaged tissue by stimulating the secretion of inflammatory cytokines [16]. Indeed, according to some studies, various processes involved in the pathogenesis of ALD, including lipid accumulation, organelle stress, hepatocyte death, immune cell activation, and fibrosis progression by hepatic stellate cells, are stimulated or promoted by oxidative stress [6]. Damage to the liver by oxidative stress can lead to the release of GOT and GPT, known as indicators of liver damage, from within hepatocytes into the blood [16,17]. In addition, data gathered from patients with advanced fibrosis or liver cirrhosis caused by ALD, serum albumin, bilirubin, and white blood cell levels are also used to determine the severity of liver injury [3]. Therefore, the effects of *A. ochotense* on chronic alcohol-induced oxidative stress and the levels of biomarkers for liver toxicity were examined (Figure 3 and Table 2). According to the results, treatment with EA extract resulted in reduced alcohol-induced lipid peroxidation, which is believed to contribute to the recovery and activity of the antioxidant system. It has also been shown that reducing oxidative stress can help improve the level of biomarkers for liver toxicity in serum. The results of a previous in vitro study on quercetin and quercetin-3-glucoside, the main bioactive substances of *A. ochotense*, showed a much higher recovery of GSH content in alcohol-induced HepG2 cells compared with rutin, and quercetin was effective in suppressing the levels of MDA [16]. One study also reported that the amount of fructose residues may have a major influence on the antioxidant potential of quercetin [16]. In addition, quercetin-3-*O*-glucoside and kaempferol-3-*O*-glucoside-rich *Erica multiflora* extract can improve the enzymatic activities of SOD, CAT, and glutathione peroxidase (GPx), as well as reduce oxidative stress by reducing the levels of MDA in Wistar rats fed high-fat and fructose diet [18]. In a hydrocortisone-induced kidney yang deficiency mouse model, treatment with *A. victorialis* aqueous extract resulted in improved kidney yang deficiency by reducing changes in an increase of MDA and a decrease of SOD [19]. In addition, the release of ALT and AST into the medium in ethanol-induced HepG2 cells was significantly inhibited by quercetin, quercetin-3-glucoside, and rutin, major components of *A. ochotense* [16]. Based on these results, the suppression of lipid peroxidation could occur through activation of the antioxidant system of various bioactive substances included in EA. In addition, our findings suggest that this will be useful in the management of oxidative stress and liver toxicity induced by chronic alcohol and prevent it from triggering and worsening various pathogenic mechanisms in ALD.

Consumption of alcohol can cause damage to mitochondrial DNA, lipid accumulation, oxidative stress, and apoptosis of hepatocytes, and it can cause changes in mitochondrial morphology and function [20]. In particular, in ALD, excessive oxidative stress can occur due to an imbalance in the antioxidant system, leading to mitochondrial destruction and dysfunction [21]. Chronic use of alcohol can lead to upregulated production of CYP2E1, leading to an increase in the concentration of acetaldehyde as well as reduced activity of ALDH, reducing oxidation of acetaldehyde and increasing accumulation, causing direct damage to the mitochondria and microtubules of hepatocytes in the liver [3]. In addition, mitochondrial overproduction of ROS resulting from increased levels of CYP2E1 and excessive levels of NADH produced by alcohol and ADH lead to a reduction in the electron transport chain and promote the formation of superoxide anion [22]. Oxidative modifications of hepatic mitochondrial protein thiols resulting from chronic consumption of alcohol can ultimately cause impairment of mitochondrial membrane polarization, which is essential for the synthesis of ATP by mitochondria [15]. Ultimately, damage to mitochondrial DNA and proteins can occur in response to upregulated levels of ROS, with a reduction of mitochondrial ATP [22]. Therefore, in this study, we examined the effect of *A. ochotense* on chronic alcohol-induced mitochondrial dysfunction (Figure 4). Treatment with EA resulted in improved levels of alcohol-induced mitochondrial ROS and restored mitochondrial dysfunction by increasing the levels of MMP and ATP. Previous studies have reported that cranberry peel, which contains flavanols including quercetin 3-glucoside, phenolic acids, and procyanidins, can prevent mitochondrial damage by improving ROS production and structural stability of the mitochondrial membrane of the liver in ethanol-induced rats [23]. In addition, one study reported that quercetin can be helpful in maintaining mitochondrial homeostasis by reducing the production of mitochondrial ROS and restoring mitochondrial membrane potential in ethanol-induced hepatocytes [24]. In brain microvascular endothelial cells subjected to oxygen-glucose deprivation, excessive production of ROS was ameliorated by ferulic acid, and mitochondrial dysfunction was improved by the reversal of decreased ATP and the levels of mitochondrial membrane potential [25]. Therefore, our findings suggest that EA can be effective in improving chronic alcohol-induced mitochondrial dysfunction by inhibiting the production of mitochondrial ROS and increasing the levels of MMP and ATP.

Accumulation of lipids in liver cells can be classified as an early alcoholic disease, and if not controlled, can develop into alcoholic steatohepatitis and alcoholic hepatitis [6]. As mentioned previously, acetaldehyde and oxidative stress generated during alcohol metabolism can be a cause of lipid metabolism disorders [4]. In particular, alcohol consumption can inhibit the phosphorylation of AMPK, a key regulator of lipid metabolism, and increase the expression of SREBPs [4,26]. In liver tissue, SREBPs are transcription factors that regulate genes related to the synthesis of cholesterol, fatty acids, and triglycerides [26]. Among them, SREBP-1 has been reported to increase in a dose-dependent manner by acetaldehyde treatment at the cellular level and regulates the transcription of genes involved in triglyceride synthesis such as FAS and stearoyl-CoA desaturase [4,26]. According to a recent study, PPAR-γ, which is involved in the storage of fatty acid and regulation of glucose metabolism, is reported to be a factor that can enhance the lipogenic action of SREBP-1c [5,27]. As a result, inhibition of AMPK phosphorylation by chronic alcohol consumption increases SREBP-1, PPAR-γ, and FAS expression, contributing to the development of hepatic steatosis, so AMPK can be considered a focus for the treatment of AFLD [4]. AFLD is typically characterized by increased levels of TG in serum and liver and/or increased fat droplets in liver tissue [28]. The levels of TG and TCHO, which are also major metabolic biomarkers in blood lipid metabolism, can be used when determining the degree of liver damage [29]. In addition, consumption of ethanol can increase the level of serum cholesterol, resulting in the accumulation of lipoproteins including VLDL- and LDL-cholesterol in the blood, with the development of lipoprotein metabolism disorders [15]. By contrast, the level of HDL-cholesterol, which has an important association with cardiovascular health, can be reduced due to alcoholic liver damage [29]. Therefore, we examined the effect of *A. ochotense* on alcohol-induced lipid metabolism disorders in serum and liver tissue (Table 2 and Figure 6). According to the results, compared with the alcohol group, the EA group showed improvement in lipid metabolism disorders in serum (Table 2). Treatment with EA resulted in increased expression of biomarkers for alcohol-induced lipid metabolism disorder (Figure 6). *A. victorialis* induced significantly increased expression of AMPK, significantly inhibited expression of SREBP-1, increased expression of PPAR-α, and decreased expression of PPAR-γ compared with the ethanol group, which ultimately improved the imbalance of lipid metabolism enzymes induced by ethanol [5]. In addition, raffinose isolated from *Costus speciosus* ethanolic water extract inhibited lipid accumulation due to a partial effect on PPAR-γ and PPAR-α mediated oxidation of fatty acid. It was also reported to suppress genes involved in adipogenesis in HepG2 fatty liver cells and 3T3-L1 adipocytes [30]. Previous studies have reported on the hepatoprotective properties of *Allium* species due to the effect of reduced levels of serum lipids and total cholesterol [31]. Based on these results, treatment with EA resulted in an improvement of the lipid metabolism imbalance caused by chronic alcohol by regulating the AMPK pathway related to levels of protein expression and regulating lipid levels in serum, which might ultimately be helpful in preventing AFLD.

Association of activation of Kupffer cells, hepatic macrophages, with chronic ALD has been reported [2]. Excessive consumption of ethanol can increase intestinal permeability and increase the movement of LPS from the intestine to the liver [1]. LPS is recognized by receptors on the surface of Kupffer cells including CD14 and TLR-4 [1]. In this process, Kupffer cells are activated, which causes activation of NF-κB and induces the production of pro-inflammatory cytokines in the liver. In addition, activation of MyD88, which connects TLR-4 and NF-κB, causes the decomposition of IκB and activates NF-κB, which is transferred from the cytoplasm into the nucleus [4], inducing transcription of pro-inflammatory cytokines such as TNF-α, IL-1β, and IL-6 [32]. Caspase 1 is a protease associated with inflammatory responses, which produces mature IL-1β and IL-18 [5]. This LPS/TLR-4 pathway can induce an inflammatory response in ALD and stimulate signaling pathways related to the activation of hepatic stellate cells (HSCs), leading to the development of alcoholic liver fibrosis [33]. In addition, oxidative stress and activation of inflammatory cells can influence each other, and in the case of alcohol-induced injury in the liver, higher levels of ROS and MDA promote the production of pro-inflammatory mediators [6,16]. Therefore, we examined the effect of *A. ochotense* on excessive production of alcohol-induced inflammatory cytokines (Figure 7). EA improved the TLR-4/MyD88/NF-kB pathway, which inhibited the expression level of pro-inflammatory cytokines. Similar to these results, *A. victorialis* regulated the expression of IL-6, caspase 1, IL-1R1, IL-1β, and TNF-α against ethanol-induced liver inflammatory response [5]. Treatment with *Erica multiflora* extract, rich in quercetin-3-*O*-glucoside and kaempferol-3-*O*-glucoside, resulted in significantly improved concentrations of plasma TNF-α and IL-6 in rats induced on a high-fat fructose diet [18]. In addition, kaempferol, one of the main bioactive substances in EA, reduced the levels of NF-κB in the nucleus in oleic acid-induced HepG2 cells and in rats fed a high-fat diet [34]. Therefore, based on these research results, treatment with EA was helpful in preventing the development of alcoholic steatohepatitis and alcoholic hepatitis, which are considered poorer prognoses than alcoholic fatty liver, through suppression of excessive immune responses induced by alcohol.

Alcohol-induced oxidative stress and programmed cell death caused by the innate immune response are believed to play a central role in the progression of ALD damage [35]. Consumption of alcohol can cause accumulation of ROS and overproduction in liver cells, leading to cell death by apoptosis and cell necrosis [14]. Another study reported on the potential for involvement of excessive alcohol-induced inflammation in apoptosis and that downstream apoptotic signaling pathways can be activated by inflammatory mediators, causing cell damage and death [36]. In particular, mitochondrial depletion of GSH is a characteristic of alcohol-dependent patients, which can impair hepatocyte resistance to TNF-α, increasing the likelihood of apoptosis [22]. In addition, ROS generated from CYP2E1 can promote activation of c-Jun N-terminal kinase (JNK) by continuous expression of the activator protein 1 (AP-1) transcription factor, leading to lipid peroxidation [15,22]. Additionally, it is reported that JNK, which is phosphorylated by various stresses such as inflammatory stimuli and ROS, phosphorylates pro-apoptotic proteins such as BCl-2 interacting mediator of cell death (BIM) protein, which activates BAX through inactivation of BCl-2 in the mitochondrial intrinsic apoptosis pathway [37]. In particular, disruption of the BAX/BCl-2 balance in the mitochondrial membrane can promote the release of cytochrome C into the cytoplasm, and cleaved caspase 3 and caspase 9 can be used as reliable indicators to determine the severity of cell death [11,14]. By contrast, activated Akt can phosphorylate BAD, BCl-2, which has an association with the anti-apoptosis protein, thus BCl-2 can inhibit BAX-induced apoptosis [37]. Therefore, in this study, we examined the question of whether treatment with *A. ochotense* would lead to the discovery of a mechanism of improvement against alcohol-induced apoptosis (Figure 8). EA was found to improve the BAX/BCl-2 ratio by regulating the JNK/Akt pathway. Similar to these results, preliminary in vitro studies have shown that the expression levels of BAX, BCl-2, and caspase were increased by treatment with *A. ochotense* inhibited apoptosis in alcohol-induced HepG2 cells [11]. Expression of alcohol-induced pro-apoptotic protein BAX was reduced by methyl ferulic acid, and the BAX/BCl-2 ratio was significantly improved. According to the results, it also suppressed apoptosis and improved anti-apoptosis by regulating the expression of ERK/MAPK/JNK protein in the liver [14]. In the seminal vesicles of rats with diabetes mellitus, quercetin was reported to exert a strong anti-apoptotic effect by reducing the expression of cleaved-caspase 3 and helping to increase the BCl-2/BAX ratio [38]. Based on these results, the BAX/BCl-2 ratio can be improved by alcohol-induced hepatocyte apoptosis through regulation of the JNK/Akt pathway and suppression of apoptosis and can enhance anti-apoptotic mechanisms by reducing the level of cleaved caspase 3.

HSCs are key cells responsible for the synthesis of collagen and have been reported as a cause of alcoholic liver fibrosis and liver cirrhosis [12]. HSCs are activated by fibrotic stimuli such as an increase in transforming growth factor beta 1 (TGF-β1), one of the most important fibrotic cytokines to date [39]. In addition, HSCs activated from inflammatory cells in sinusoidal blood vessels differentiate into myofibroblasts, migrate to the damaged area, and exhibit the characteristic of expressing fibrotic genes such as alpha-1 smooth muscle actin and chollagen-1 [2]. TGF-β1 phosphorylates Smad 2 and Smad 3, and the phosphorylated Smad2/3 complex moves to the nucleus with Smad 4, activating the expression of TGF-β target genes including collagen type 1 alpha 1 gene (COL1A1) [39]. Furthermore, with the continuation of the vicious cycle in ALD patients, acetaldehyde-protein adducts are formed, disabling DNA repair, damaging mitochondria in hepatocytes, and further promoting the synthesis and deposition of collagen bands between central veins and veins [3]. Continuous and long-term activation of HSCs and myofibroblasts can result in progressive deposition of collagen, leading to the development of liver fibrosis and, in advanced stages of liver fibrosis, cirrhosis [2]. This type of liver fibrosis is a temporary and reversible reaction, and in some patients, the condition will return to normal when alcohol consumption is stopped. However, continued drinking can lead to chronic inflammation and excessive fibrosis, with the replacement of liver parenchyma with scar tissue, causing serious damage to the liver [1]. Therefore, in this study, we examined the effect of *A. ochotense* on alcohol-induced liver fibrosis (Figure 9). EA decreased the expression levels of alcohol-induced TGF-β1 and p-Smad2/3 and appeared to improve the expression level of α-SMA. Similarly, *A. victorialis* inhibited the expression of TGF-β1 mRNA in high-sugar-induced mouse kidney mesangial cells (MMCs), and among the eight compounds identified, ferulic acid showed the greatest inhibitory effect on the expression of TGF-β1 in MMC [40]. Ferulic acid has also been reported to inhibit the activation of HSCs by blocking TGF-β signaling by HSC-T6 cells and reducing ERK1/2 and Smad signaling [41]. Quercetin scavenged ROS, attenuated TGF-β1/Smad 3 signaling, and stimulated Nrf-2 and Smad 7. A protective effect on myocardial infarction-induced left ventricular remodeling and fibrosis through antioxidant, anti-inflammatory, and anti-fibrotic effects was observed [42]. Based on these results, treatment with *A. ochotense* was able to reduce the formation of phosphorylated Smad2/3 complexes by improving TGF-β1 expression induced by inflammation and oxidative stress. This ultimately prevented the differentiation of myofibroblasts and is thought to have a protective effect against alcohol-induced liver fibrosis by reducing fibrosis genes such as α-SMA.

## 4. Materials and Methods

### 4.1. Sample Preparation

*A. ochotense* grown at a farm (Sancheong, Republic of Korea) was obtained by the Gyeongsangnam-do Forest Environment Research Institute (Jinju, Republic of Korea) in May 2022. *A. ochotense* leaves were washed, lyophilized with a vacuum drier (Operon, Gimpo, Republic of Korea), and powdered. Distilled water and 60% ethanol were added, respectively, to *A. ochontense* powder at a ratio of 1:50 (*w*/*v*) and extracted at 40 °C for 2 h and then filtered using a 0.45 μm filter (Whatman Inc., Kent, UK), lyophilized, and stored at −20 °C until the experiment was performed.

### 4.2. Identification of Bioactive Substance in Allium ochotense

The mixture of *A. ochotense* extract was dissolved in 50% methanol and filtered through a 0.2 µm filter (Whatman Inc., Kent, UK). This was identified using the Waters Acquity ultra-performance liquid chromatography-quadrupole time of flight mass spectrometry (UPLC QTOF/MS^E^) system (UPLC-Q-TOF/MS^E^, Vion, Waters Corp., Milford, MA, USA). Physiological compounds in *A. ochotense* were separated by Acquity UPLC BEH C18 column (2.1 × 100 mm, 1.7 μm pore, Waters Corp, Milford, MA, USA). Solvent (mobile phase A: distilled water with 0.1% formic acid and B: methanol) gradient was added as follows: 0 to 0.5 min, 0%; 0.5 to 8 min, 0 to 100%; 8 to 8.5 min, 100%; 8.5 to 10 min, 100 to 0%; and 10 to 11 min, 0%. Ionization was performed by electrospray ionization (ESI) and was performed in negative ion mode. Additionally, the MS analysis conditions were as follows: drying gas (N_2_) temperature 350 °C; ramp collision energy, 10–30 V; oven temperature, 40 °C; drying gas flow 30 L/h, capillary voltage 3 kV, pressure of nebulizer, 40 psi; fragmented voltage 175 V, cone voltage, 40 V; and mass range from 50 to 1200 *m*/*z*.

### 4.3. Animal Experimental Design

#### 4.3.1. In Vivo Alcohol Degradation Experimental Model

For the establishment of an animal model for evaluating in vivo alcohol-degrading metabolic ability, C57BL/6 (four weeks old, male) mice were purchased from a laboratory animal supplier (Samtako, Osan, Republic of Korea). There were eight groups, and the experimental animals were randomly divided into three mice per cage (*n* = 3) and adapted to the environment for seven days. Experimental animals were raised in a consistently maintained environment where the temperature (22 ± 2 °C) and humidity (55%) were constant, and adequate drinking water and feed were supplied under 12 h light/dark conditions. Drinking water was administered to experimental animals that were adapted for a week in the control and alcohol groups, and dried powder of *A. ochotense* was dissolved in drinking water for treatment in the sample group once a day. The *A. ochotense* treatment group was treated with aqueous extract (0 L and 0 H, respectively), 60% ethanol extract (60 L and 60 H, respectively), and a mixture of *A. ochotense* extracts (mixing ratio of aqueous and 60% ethanol extracts 2:8 (*w*/*w*), 2:8 L and 2:8 H, respectively). The extract treatment group was divided into low-concentration and high-concentration, with oral administration (low-concentration (L), 50 mg/kg of B.W.; high-concentration (H), 100 mg/kg of B.W.). After 30 min, 25% alcohol (5 g/kg of B.W.) was administered to the remaining experimental groups, excluding the control group.

Following oral administration of 25% alcohol, the experimental animals were sacrificed sequentially at 0 min, 30 min, 60 min, 120 min, and 180 min, and blood samples were obtained from the abdominal vein. Blood samples obtained for each period were centrifuged at 13,000× *g* for 10 min at 4 °C, and the supernatant serum was used as an analysis sample. Serum alcohol and acetaldehyde concentrations were measured using commercial kits (Megazyme Int., Bray, Ireland). AUC was measured using the trapezoidal method (for the time interval of the obtained blood samples) [43,44].

#### 4.3.2. Alcohol-Induced Fatty Liver Experimental Model

C57BL/6 (four weeks old, male) mice were purchased from a laboratory animal supplier and adapted to the surrounding environment for seven days. Temperatures (22 ± 2 °C) and humidity (55%) were kept constant, and adequate drinking water and feed were supplied to the experimental animals under environmental conditions of 12 h of light/dark. After adaptation for one week, the samples were administered orally to the experimental animals. There were five groups, and the experimental animals were randomly divided into 15 mice per group (ex vivo test, *n* = 7; serum analysis, mitochondrial, *n* = 5; western blot assay, *n* = 3) and placed in cages. The control and alcohol groups received the same amount of drinking water instead of the samples, and the mixture of *A. ochotense* extract was dissolved in drinking water and administered orally for 8 weeks. The mixture was prepared by mixing water extract and 60% ethanol extract at a mixing ratio of 2:8 and used in the experiment. The NS group was treated with a mixture of aqueous and 60% ethanol extracts of *Allium ochotense* (100 mg/kg of B.W.). The EA50 and EA100 groups were treated with a mixture of aqueous and 60% ethanol extract of *Allium ochotense* at concentrations of 50 and 100 mg/kg of B.W, respectively. Furthermore, 30 min after oral administration of the sample, 25% alcohol (5 g/kg of body weight) was administered orally to all groups except the control and NS groups. The control and NS groups were provided drinking water the same way. After eight weeks of treatment with sample and alcohol, experimental animals were sacrificed for serum analysis and evaluation of fatty liver-related biomarkers, and blood was collected from the abdominal vena cava and liver was obtained [43].

All animal experiments were performed in accordance with the Institutional Animal Care and Use Committee of Gyeongsang National University (Certificate: GNU-221103-M0155-01, Approval date: 31 March 2023).

### 4.4. Measurement of Fatty Liver and Hepatotoxicity Biomarkers in Serum

Blood samples collected were centrifuged at 13,000× *g* for 10 min at 4 °C, and the supernatant serum was used as an analysis sample. The TCHO, TG, and HDL-cholesterol concentrations of the obtained serum samples were measured with an automatic chemistry analyzer (DRI-CHEM 4000i, FUGIFILM, Tokyo, Japan). And LDL-cholesterol was calculated as calculated as follows:

LDLC (mg/dL) = TCHO-(HDLC + TG/5)(1)

To evaluate hepatotoxicity, liver damage indicators GOT, GPT, and TBIL were measured using an automatic chemistry analyzer (DRI-CHEM 4000i, FUGIFILM, Tokyo, Japan) [45].

### 4.5. Antioxidant System

#### 4.5.1. Preparation of Tissue

To analyze the levels of MDA and SOD in liver tissue, it was homogenized using a bullet blender (Next Advance Inc., Raymertown, NY, USA) after adding the 10-fold volume of phosphate buffered saline (PBS, pH 7.4). In addition, to investigate reduced GSH content, 10 mM phosphate buffer with 1 mM ethylenediaminetetraacetic acid (EDTA) corresponding to a 10-fold volume of liver tissue was added and homogenized. The protein concentration of the homogenized and centrifuged samples was calculated according to the Bradford method [43].

#### 4.5.2. Measurement of Antioxidant System Biomarkers

To measure the MDA contents, the liver homogenate was centrifuged at 2356× *g* for 10 min at 4 °C. Then, 1% phosphoric acid and 0.67% thiobarbituric acid were added to the obtained supernatant and reacted at 95 °C for 1 h. The reaction solution was centrifuged at 2356× *g* for 10 min at 4 °C. The absorbance of the supernatant was measured at 532 nm, and the MDA content was expressed as nmole/mg of protein content [46].

To evaluate the SOD content in liver tissue, it was centrifuged at 400× *g* for 30 min at 4 °C, and the supernatant was removed. The 1 × Cell extraction buffer (10 × SOD buffer, 20% trition X, distilled water, and 200 mM phenylmethylsulfonyl fluoride (pMSF)) was added to the separated pellet. Then, the mixture reacted on ice for 30 min. After 30 min, the reaction solution was centrifuged at 10,000× *g* for 10 min at 4 °C. The supernatant was evaluated using a SOD kit (Dojindo, Kumamoto, Japan), and the SOD level was expressed as U/mg of protein.

To investigate the reduced GSH content in liver tissue, the homogenate was centrifuged at 10,000× *g* for 15 min at 4 °C, and the supernatant was obtained. The same amount of 5% metaphosphoric acid was added to the supernatant, and the interfering proteins were removed by spinning down at 2000× *g* for 2 min at 4 °C. Then, the obtained supernatant was mixed with 0.26 M tris-HCl (pH 7.8), 0.65 N NaOH, and 2 mg/mL of *o*-phthalaldehyde (dissolved in methanol) for 15 min at room temperature in a dark place. After 15 min of reaction, the fluorescence of the solution was measured at wavelengths of 320 nm (excitation) and 420 nm (emission) at 1 min intervals using a fluorometer (Infinite F200, Tecan, Männedorf, Switzerland). The content of reduced GSH is expressed as % of control [46].

### 4.6. Mitochondrial Function

#### 4.6.1. Extraction of Mitochondrial Sample

To isolate mitochondria, mitochondrial isolation (MI) buffer (215 mM mannitol, 75 mM sucrose, 0.1% bovine serum albumin (BSA), 20 mM hydroxyethyl piperazine ethane sulfonic acid (HEPES) sodium salt, pH 7.2) containing 1 mM ethylene glycol-bis(β-aminoethyl ether)-*N*,*N*,*N*′,*N*′-tetraacetic acid (EGTA) was added to liver tissue and homogenized. The homogenate was centrifuged at 1300× *g* for 5 min at 4 °C. Then, the obtained supernatant was centrifuged again at 13,000× *g* for 10 min at 4 °C. MI buffer containing 1 mM EGTA and 0.1% digitonin was added to the pellet and reacted on ice for 5 min. After 5 min of reaction, the reaction solution was centrifuged at 13,000× *g* for 15 min at 4 °C. The final pellet was mixed again with MI buffer and the mixture was used for the mitochondrial experiment [47].

#### 4.6.2. Measurement of Mitochondrial Function Biomarkers

To investigate the mitochondrial ROS level, the extracted liver mitochondrial sample was mixed with respiration buffer (125 mM KCl, 2 mM KH_2_PO_4_, 20 mM HEPES, 1 mM MgCl_2_, 500 μM EGTA, 2.5 mM malate, and 5 mM pyruvate) and 25 μM 2′,7′-dichlorofluorescein diacetate (DCF-DA). The mixture was incubated for 20 min in a dark place. Then, the fluorescence was measured at wavelengths of 485 nm (excitation) and 535 nm (emission) using a fluorometer (Infinite F200, Tecan, Männedorf, Switzerland) [47,48].

To evaluate the levels of mitochondrial membrane potential, the mitochondrial extract samples were mixed with MI buffer with 5 mM pyruvate, 5 mM malate, and 1μM JC-1. The mixture was incubated in a dark place at room temperature for 20 min and mixed gently at 5 min intervals. After 20 min of incubation, the fluorescence of the reaction solution was measured at wavelengths of 530 nm (excitation) and 590 nm (emission) using a fluorometer (Infinite F200, Tecan, Männedorf, Switzerland) [47].

To investigate the mitochondrial ATP level, the mitochondrial extract was measured using the commercial ATP assay kit (Sigma-Aldrich Chemical Co., Milwaukee, WI, USA) and a luminometer (Glomax^®^, Promega, Sunnyvale, CA, USA).

### 4.7. Investigation of Protein Expression Levels in Liver Tissue Using Western Blot Assay

To evaluate the protein expression levels, the liver tissue was homogenized with a 10-fold volume of the protein extraction buffer (Gene All Biotechnology, Seoul, Republic of Korea) containing 1% protease inhibitor (Quartett, Berlin, Germany). The mixture was centrifuged at 13,000× *g* for 10 min at 4 °C. Then, the obtained supernatant was mixed with sample loading buffer (#1610374, Bio-Rad, Richmond, CA, USA) and used as a western blot sample. The immunoblotting analysis sample was separated by sodium dodecyl sulfate-polyacrylamide gel electrophoresis (SDS-PAGE) and transferred to a PVDF membrane (Millipore, Billerica, MA, USA). Afterwards, the membrane was treated with 5% skim to block for 1 h. Then, the membrane reacted with the primary antibody at 4 °C for 12 h. The secondary antibody was treated with the membrane at room temperature for 1 h. This antibody complex was reacted with ECL solution to develop color and detected using an image analyzer (iBright ^TM^ CL1000, Invitrogen, Waltham, MA, USA). The expression levels of detected proteins were calculated using ImageJ software (ImageJ 1.53t, National Institutes of Health, Bethesda, MD, USA). In addition, the antibody information used in this study is shown in Table 3.

### 4.8. Statistical Analysis

All data were expressed as mean ± SD. Significant differences between groups were calculated and displayed using one-way analysis of variance (ANOVA). Significant differences were confirmed using Duncan’s new multiple range test (*p* < 0.05) method through the SAS program (version 9.4, SAS Institute Inc., Cary, NC, USA). Statistically significant differences are indicated with different lowercase letters.

## 5. Conclusions

**Figure 10:**
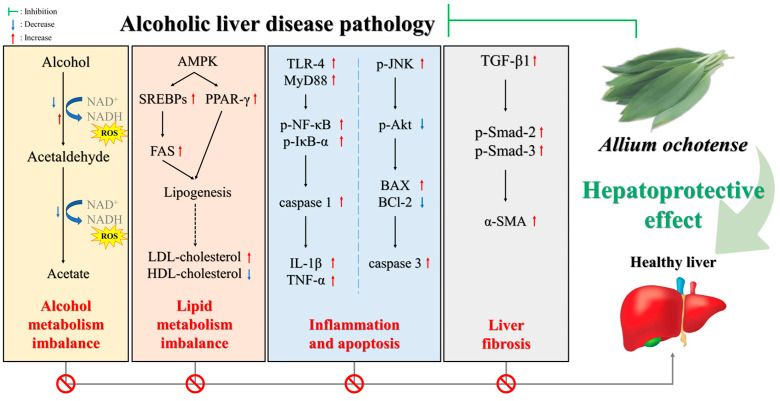
A scheme of improvement mechanism of *Allium ochotense* on alcoholic liver disease pathology.

In this study, it was confirmed that *A. ochotense* improves AFLD by improving lipid metabolism disorders and liver inflammation. EA helped improve alcohol and acetaldehyde degradation ability, thereby improving blood alcohol concentration and acetaldehyde concentration. The regulation of alcohol-induced lipid metabolism imbalance and hepatotoxicity biomarkers was probably restored by the treatment of EA against improving alcohol degradation ability. Also, treatment with EA inhibited MDA formation by activating the antioxidant biomarker such as SOD and reduced GSH, suppressed ROS production, and increased levels of MMP and ATP, helping to restore mitochondrial function. In addition, EA increased the expression levels of alcohol-degrading enzymes, which helped improve alcohol-induced lipid metabolism disorders. In particular, it was able to protect apoptosis and liver fibrosis from the improvement of overexpression of alcoholic hepatitis factors. In addition, it is considered that the various protective mechanisms of EA on the chronic alcohol-induced liver may be due to bioactive substances such as kestose, raffinose, kaempferol di-glucoside, quercetin glucoside, and kaempferol glucoside isomer. In conclusion, these results suggest that EA can be used as a natural high-value-added material that can prevent fatty liver disease by improving alcohol-induced lipid metabolism disorders and alcoholic hepatitis (Figure 10). However, additional study is needed on the synergistic effects of the mixture and the detailed effects on the main bioactive substances.

## Figures and Tables

**Table 1:** Identification of main compounds of the aqueous extract of the mixture of the aqueous and 60% ethanol (2:8, *w*/*w*) extracts of *Allium ochotense* (EA) using UPLC-Q-TOF/MS^E^ chromatography.

No.	RT (min)	*m*/*z* [M − H]^−^	Fragment (*m*/*z*)	Proposed Compounds
1	0.79	503	179, 341, 101, 323, 221, 113	Ketose
2	1.21	503	179, 341, 323, 221, 179	Raffinose
3	3.65	609	447, 285	Kaempferol di-glucoside isomer
4	3.91	463	301, 285, 464	Quercetin glucoside isomer
5	4.28	447	174, 285, 229	Kaempferol glucoside isomer

RT: retention time.

**Table 2:** Ameliorating effects of the mixture of aqueous and 60% ethanol (2:8, *w*/*w*) extracts of *Allium ochotense* (EA) on liver toxicity and fatty liver related to the biomarkers in serum.

	TCHO (mg/dL)	TG (mg/dL)	HDLC (mg/dL)	LDLC (mg/dL)	GOT (U/L)	GPT (U/L)	TBIL (mg/dL)
Control	94.50 ± 3.42 ^c^	115.50 ± 5.20 ^b^	74.00 ± 4.16 ^bc^	3.45 ± 1.59 ^b^	45.40 ± 4.45 ^b^	29.40 ± 1.52 ^b^	0.50 ± 0.07 ^b^
NS	95.75 ± 7.89 ^c^	101.75 ± 12.66 ^b^	76.25 ± 2.06 ^bc^	4.00 ± 0.59 ^b^	44.80 ± 1.30 ^b^	29.20 ± 4.76 ^b^	0.52 ± 0.22 ^b^
Alcohol	110.25 ± 1.50 ^a^	143.50 ± 12.50 ^a^	70.50 ± 3.70 ^c^	11.40 ± 3.98 ^a^	54.00 ± 10.12 ^a^	35.40 ± 4.92 ^a^	0.70 ± 0.07 ^a^
EA50	97.25 ± 5.68 ^bc^	104.25 ± 16.94 ^b^	82.25 ± 11.90 ^ab^	0.90 ± 4.67 ^b^	47.40 ± 2.30 ^ab^	34.20 ± 4.09 ^a^	0.38 ± 0.04 ^b^
EA100	105.00 ± 9.70 ^ab^	113.25 ± 2.50 ^b^	91.00 ± 4.76 ^a^	0.55 ± 0.91 ^b^	46.00 ± 2.00 ^b^	29.40 ± 1.82 ^b^	0.42 ± 0.04 ^b^

TCHO, total cholesterol; TG, triglyceride; HDLC, high density lipoprotein cholesterol; LDLC, low density lipoprotein cholesterol; GOT, glutamic oxaloacetic transaminase; GPT, glutamic pyruvic transaminase; TBIL, total bilirubin. NS group, the mixture of aqueous and 60% ethanol extracts of *Allium ochotense* (100 mg/kg of B.W.) + drinking water; EA50 group, the mixture of aqueous and 60% ethanol extracts of *Allium ochotense* (50 mg/kg of B.W.) + alcohol; EA100 group, the mixture of aqueous and 60% ethanol extracts of *Allium ochotense* (100 mg/kg of B.W.) + alcohol. The results are shown as mean ± SD (serum analysis, *n* = 5). Data were statistically considered at *p* < 0.05, and different lowercase letters indicate statistical differences.

**Table 3:** List of the antibody information used in this study.

Antibody	Catalog No.	Concentration	Manufacturer
β-actin	sc-69879	1:1000	Santa Cruz Biotechnology (Dallas, TX, USA)
ADH	sc-133207	1:1000	Santa Cruz Biotechnology (Dallas, TX, USA)
ALDH	sc-374076	1:1000	Santa Cruz Biotechnology (Dallas, TX, USA)
p-AMPK	sc-33524	1:1000	Santa Cruz Biotechnology (Dallas, TX, USA)
SREBP-1	sc-13551	1:1000	Santa Cruz Biotechnology (Dallas, TX, USA)
PPAR-γ	sc-7273	1:1000	Santa Cruz Biotechnology (Dallas, TX, USA)
p-JNK	sc-6254	1:1000	Santa Cruz Biotechnology (Dallas, TX, USA)
p-Akt 1/2/3	sc-393887	1:1000	Santa Cruz Biotechnology (Dallas, TX, USA)
BAX	sc-7480	1:1000	Santa Cruz Biotechnology (Dallas, TX, USA)
BCl-2	sc-509	1:1000	Santa Cruz Biotechnology (Dallas, TX, USA)
caspase 3	sc-56053	1:1000	Santa Cruz Biotechnology (Dallas, TX, USA)
TLR-4	sc-52962	1:1000	Santa Cruz Biotechnology (Dallas, TX, USA)
MyD88	sc-74532	1:1000	Santa Cruz Biotechnology (Dallas, TX, USA)
p-NF-κB	sc-136548	1:1000	Santa Cruz Biotechnology (Dallas, TX, USA)
p-IκB-α	sc-8404	1:1000	Santa Cruz Biotechnology (Dallas, TX, USA)
caspase 1	sc-392736	1:1000	Santa Cruz Biotechnology (Dallas, TX, USA)
IL-1β	sc-515598	1:1000	Santa Cruz Biotechnology (Dallas, TX, USA)
TNF-α	sc-33639	1:1000	Santa Cruz Biotechnology (Dallas, TX, USA)
TGFβ-1	sc-130348	1:1000	Santa Cruz Biotechnology (Dallas, TX, USA)
p-Smad-3	sc-517575	1:1000	Santa Cruz Biotechnology (Dallas, TX, USA)
CYP2E1	CSB-PA006425EA01HU	1:1000	Cusabio (Wuhan, China)
FAS	CSB-PA06354A0Rb	1:1000	Cusabio (Wuhan, China)
α-SMA	Ab5694	1:1000	Abcam (Cambridge, UK)
p-Smad-2	#3108	1:1000	Cell Signaling Technology (Danvers MA, USA)
Anti-rabbit	7074S	1:5000	Cell Signaling Technology (Danvers, MA, USA)
Anti-mouse	#1706516	1:5000	Bio-Rad (Richmond, CA, USA)

## Data Availability

The data presented in this study are available on request from the corresponding author.
